# A chemical screen identifies the chemotherapeutic drug topotecan as a specific inhibitor of the B-MYB/MYCN axis in neuroblastoma

**DOI:** 10.18632/oncotarget.498

**Published:** 2012-05-19

**Authors:** Francesco Sottile, Ilaria Gnemmi, Sandra Cantilena, Walter C. D'Acunto, Arturo Sala

**Affiliations:** ^1^ UCL Institute of Child Health, London, UK; ^2^ Brunel Institute of Cancer Genetics and Pharmacogenomics, Dept. of Biosciences, Heinz Wolf Building, Brunel University, Kingston Avenue, London, UK.

**Keywords:** Neuroblastoma, chemotherapeutic drug, oncogene, transcription

## Abstract

The transcription factor MycN is the prototypical neuroblastoma oncogene and a potential therapeutic target. However, its strong expression caused by gene amplification in about 30% of neuroblastoma patients is a considerable obstacle to the development of therapeutic approaches aiming at eliminating its tumourigenic activity. We have previously reported that B-Myb is essentially required for transcription of the *MYCN* amplicon and have also shown that *B-MYB* and *MYCN* are engaged in a feed forward loop promoting the survival/proliferation of neuroblastoma cells. We postulated that pharmacological strategies breaking the *B-MYB/MYCN* axis should result in clinically desirable effects. Thus, we implemented a high throughput chemical screen, using a curated library of ~1500 compounds from the National Cancer Institute, whose endpoint was the identification of small molecules that inhibited B-Myb. At the end of the screening, we found that the compounds pinafide, ellipticine and camptothecin inhibited B-Myb transcriptional activity in luciferase assays. One of the compounds, the topoisomerase-1 inhibitor camptothecin, is of considerable clinical interest since its derivatives topotecan and irinotecan are currently used as first and second line treatment agents for various types of cancer, including neuroblastoma. We found that neuroblastoma cells with amplification of *MYCN* are more sensitive than *MYCN* negative cells to camptothecin and topotecan killing. Campothecin and topotecan caused selective down-regulation of B-Myb and MycN expression in neuroblastoma cells. Notably, forced overexpression of B-Myb could antagonize the killing effect of topotecan and camptothecin, demonstrating that the transcription factor is a key target of the drugs. These results suggest that camptothecin and its analogues should be more effective in patients whose tumours feature amplification of *MYCN* and/or overexpression of *B-MYB*.

## INTRODUCTION

Neuroblastoma is a tumour of the sympathetic nervous system and the most common extracranial solid tumour in childhood. It represents more than 7% of malignancies in patients younger than 15 years and around 15% of all paediatric oncology deaths. The overall incidence is about one case in 7,000 live births, and there are about 700 new cases per year in the United States [1, 2].

Amplification of *MYCN* is the most common genetic aberration associated with poor outcome in neuroblastoma [3, 4], occurs in roughly 30% of primary tumours and is strongly correlated with advanced disease and treatment failure [5, 6]. Its association with poor outcome in patients with otherwise favourable disease features, such as localized tumours or INSS stage 4S disease, underscores its biological importance [7-9]. The *MYCN* proto-oncogene encodes a 60-63 KDa protein (MycN) that is exclusively expressed in the developing nervous system, unlike its paralogue *c-MYC*, which is expressed ubiquitously [10]. Like all Myc family proteins, MycN contains an N-terminal transactivating domain (MYC box) and a C-terminal region containing a basic-helix-loop-helix/leucine zipper (bHLH-LZ) motif, which mediates DNA binding as well as binding to other bHLH-LZ proteins such as Max and Mad [11].

*MYCN* is located on the distal short arm of chromosome 2 (2p24): a large region from this site becomes amplified and the *MYCN* locus is copied to form an extrachromosomal circular element, or DM (double-minute chromatin bodies), with retention of the normal copies of *MYCN* at 2p24. DMs might accumulate by uneven segregation during mitosis; however, in some cases, the amplified DNA integrates into a chromosomal locus to form an HSR (homogeneously staining regions) [12-15]. Other genes might be co-amplified with *MYCN* in a subset of cases, but *MYCN* is the only gene that is consistently amplified from this locus [4].

The vertebrate Mybs comprise a small family of transcription factors. The prototypical member, c-Myb, is the cellular homologue of the oncogene carried by the AMV and E26 chicken retroviruses that transform haematopoietic cells *in vivo* and *in vitro* [16]. The two other members of the family, A-Myb and B-Myb, share a similar protein domain organization and bind to the same consensus sequence on the DNA [17-19]. *B-MYB* is expressed early during mouse embryogenesis, and is associated with cell proliferation. Consistent with this, *B-MYB* knock-out mice die at a very early stage of development, and the requirement for B-Myb in inner cell mass formation is indicated by the severely impaired proliferation of these pluripotent cells when blastocysts were explanted and cultured *in vitro* [20-24]. *B-MYB* antisense oligonucleotides inhibit proliferation of normal and transformed cell lines, while constitutive *B-MYB* expression allows BALB/c 3T3 fibroblasts to grow in low serum conditions and prevents cell cycle arrest and differentiation of M1 myeloid leukaemia cells treated with interleukin (IL)-6 [25-27]. B-Myb is a relatively weak, ubiquitous transcription factor and in normal physiological settings it is not essential for the transcription of *MYCN*, whose expression is strictly tissue specific. However, in neuroblastoma the generation of multiple copies of the *MYCN* gene causes accumulation of the MycN oncoprotein, which binds to the *B-MYB* locus and activates its unregulated expression. This will initiate a pathological regulatory cycle where B-Myb, in spite of its intrinsically weak transcriptional activity, will cause a significant enhancement of *MYCN* expression due to the large number of template amplicons available [28]. Given their reliance on *B-MYB*, neuroblastoma tumours with amplification of *MYCN* should be exquisitely sensitive to its pharmacological targeting, suggesting that small molecule inhibitors of B-Myb could have important clinical applications.

## RESULTS AND DISCUSSION

### A chemical screen identifies small-molecule transcriptional inhibitors of B-Myb

**Figure 1:**
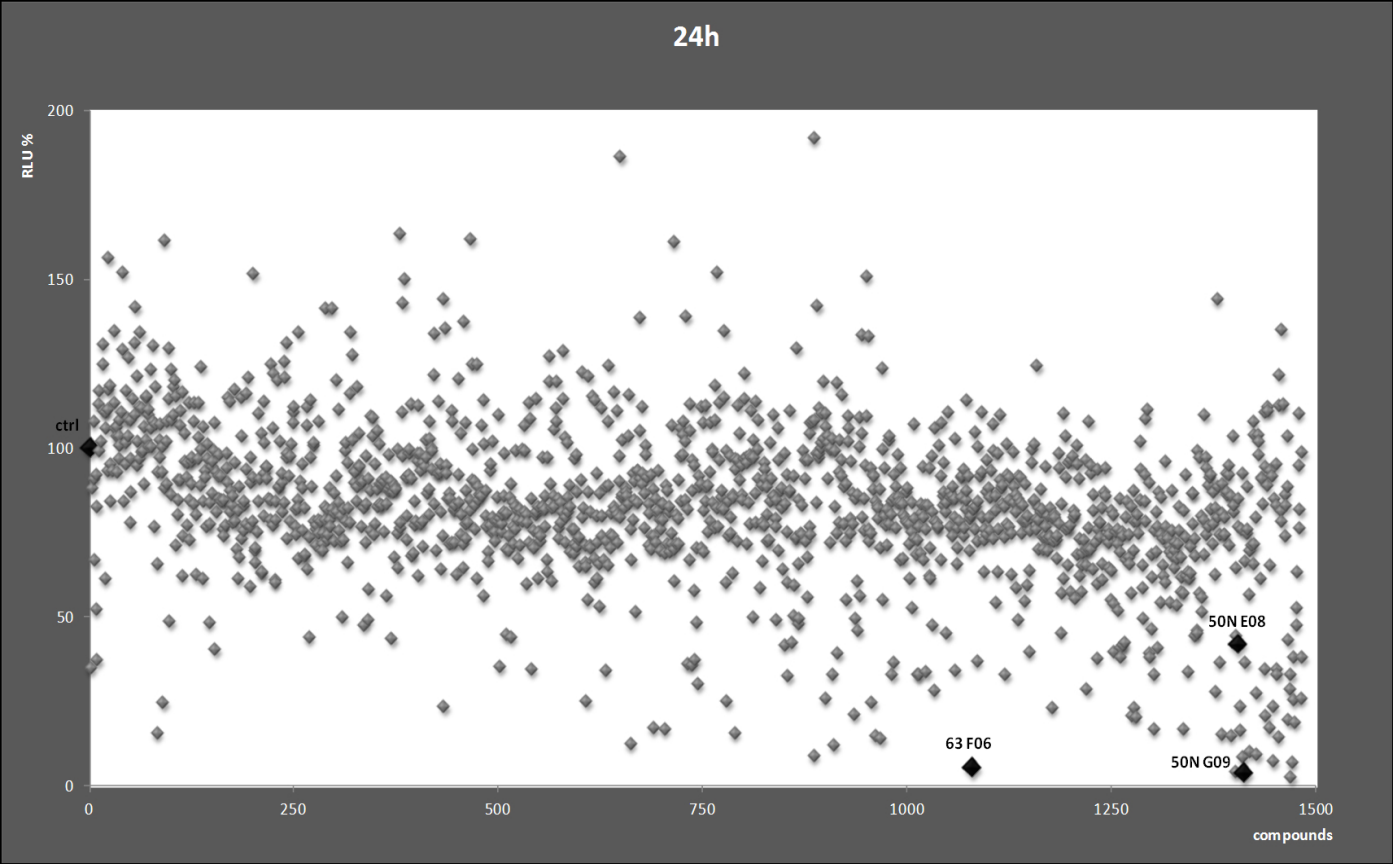
Chemical screen Each dot represents relative luciferase activity (RLU) expressed as percentages relative to the value obtained in cells treated with the vehicle DMSO (indicated by the black diamond and ctrl), which was set as 100%. The highlighted compound codes 50N E8, 50N G9, and 63 F6 indicate ellipticine, camptothecin and pinafide, respectively.

**Figure 2:**
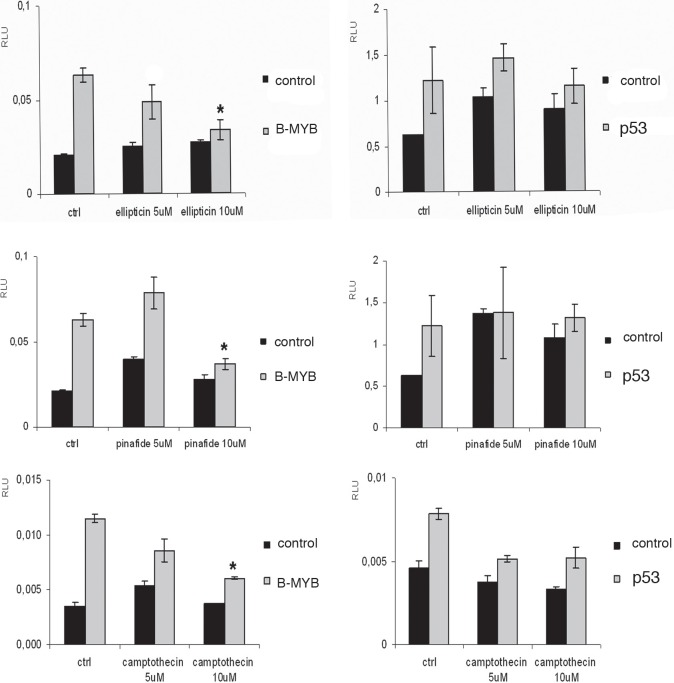
Validation of compounds identified in the primary screen GIMEN (*MYCN* non-amplified neuroblastoma cells) were transiently co-transfected with the MYB-responsive promoter pGL2-mim1 and the pcDna-*B-MYB/*empty vectors or with the p53 responsive p21luc promoter and pCMVp53/empty vectors. A renilla luciferase plasmid was added to the transfection mix for normalisation. Cells were treated with the compounds ellipticine, pinafide and camptothecin at the indicated concentrations for 24h. Error bars indicate standard deviations and the asterisk indicates statistically significant differences (Student's T test p ≤ 0,05) between the activities of cells treated with the compound relative to untreated cells (indicated by ctrl).

To identify small molecules with the potential of inhibiting B-Myb transcriptional activity and, consequently, its downstream genes such as *MYCN*, we used 2 chemical libraries from the National Cancer Institute (NCI), i.e. the diversity set II and the natural product set. Each chemical compound was assessed in a high throughput assay with a neuroblastoma cell line stably transfected with a *MYB* responsive promoter linked to the luciferase gene and a *B-MYB* expression vector. The aim was to find compounds that inhibited B-Myb transcriptional activity more than 50% compared to control. At the end of the screening, we observed that several compounds showed various degrees of inhibitory activity. Curiously, some compounds were activators of B-Myb transcriptional activity, but were not investigated further (Fig. 1). The compounds ellipticine, pinafide and camptothecin were selected for further analysis given their potential anticancer activity, and we confirmed their ability to suppress B-Myb transcriptional activity in independent transient transfection luciferase assays (Fig. 2A). As a control, we verified that the inhibitory effect on p53-mediated transactivation was weak, corroborating the hypothesis that the effect of the compounds is, at least to some extent, specific to B-Myb (Fig. 2B). Pinafide, ellipticine and camptothecin, are plant antibiotics with a vague structural similarity, all containing aromatic rings with nitrogen atoms. Camptothecin and ellipticine are known topoisomerase 1 and 2 inhibitors, respectively. They cause DNA damage and are used as chemotherapeutic drugs [29-32]. Two water-soluble derivatives of camptothecin, topotecan and irinotecan, are in use as first or second line antineoplastic agents in a variety of cancers, including neuroblastoma [32-37].

### Biological effects of the B-Myb-inhibiting compounds in neuroblastoma cell lines

**Figure 3:**
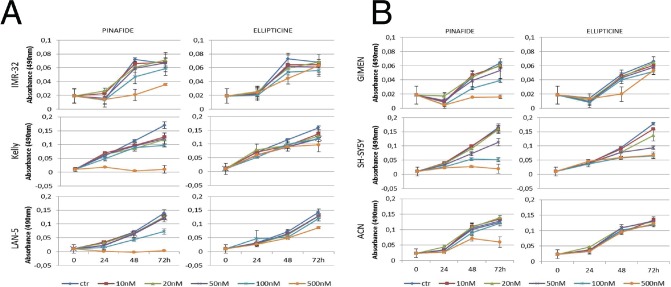
MTS proliferation assay in neuroblastoma cell lines exposed to ellipticine and pinafide Proliferation of *MYCN* amplified (panel A) or *MYCN* non amplified (panel B) cells in the presence of increasing concentrations of Ellipticine and Pinafide, as indicated. Note that cell viability is only reduced by high concentrations (250-500nM) of pinafide, in a *MYCN*-independent manner. Neuroblastoma cell lines were generally resistant to the killing effect of ellipticine. Error bars indicate standard deviations.

**Figure 4:**
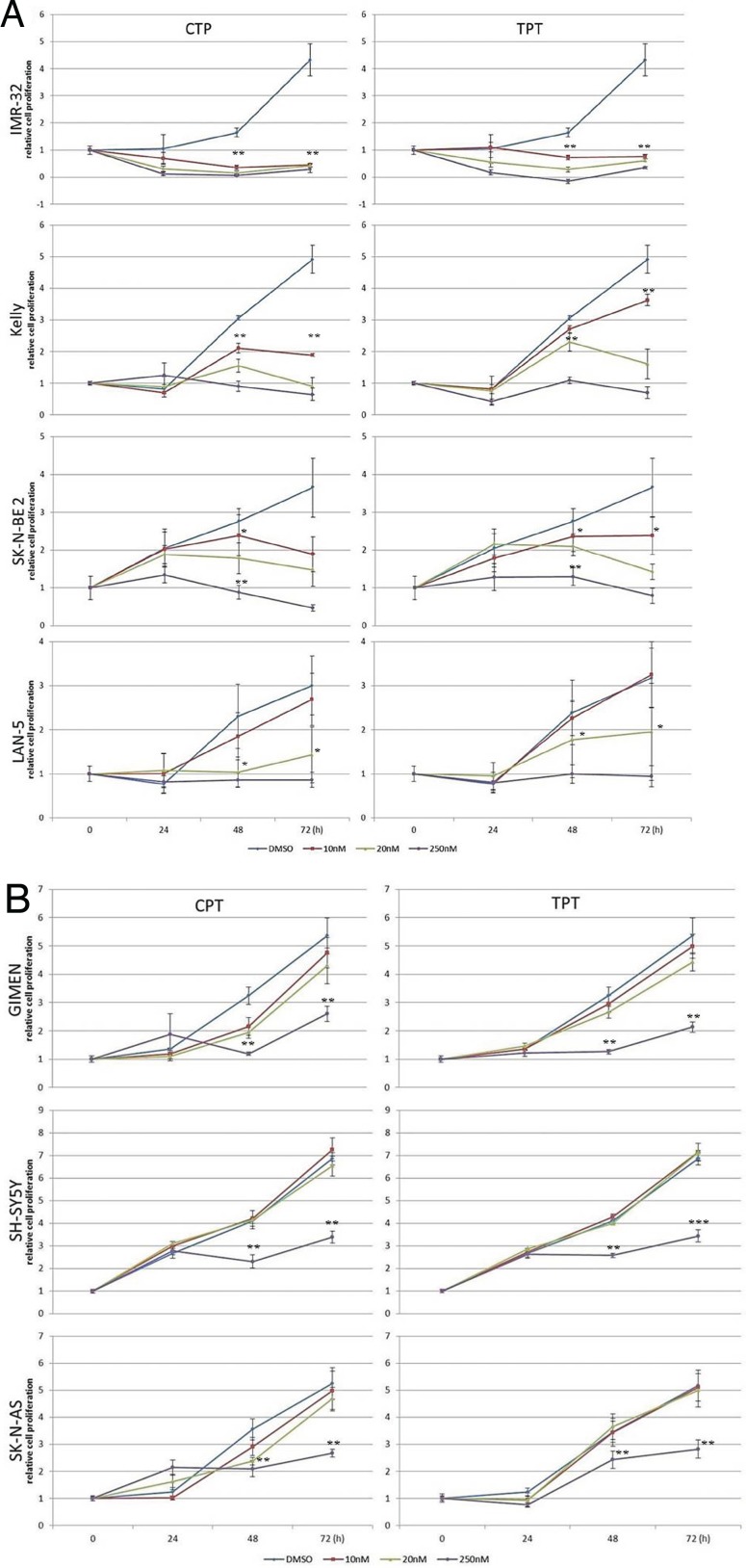
MTS proliferation assay in neuroblastoma cell lines exposed to camptothecin (CPT) or topotecan (TPT) A) *MYCN*-amplified NB cells; B) *MYCN* non-amplified NB cells. Error bars indicate standard deviations and statistical significance was verified by Student's T test (*= p<0,05 **=p<0,01 ***=p<0,001 of 48hrs 10v20v250nM vs. CTR; 72h 10v20v250nM vs. CTR )

We used camptothecin, pinafide and ellipticine in proliferation assays with a panel of *MYCN* amplified or non-amplified cell lines. Pinafide showed some killing activity at the highest concentrations used, i.e. 250-500 nM, independently from the presence of *MYCN* amplification (Fig. 3A,B). Neuroblastoma cell lines were resistant to escalating doses of ellipticine up to a concentration of 500nM, with the exception of SH-SY5Y cells which were inhibited by high concentrations of the drug (Fig. 3B). Notably, neuroblastoma cell lines with amplification of *MYCN* were extremely sensitive to concentrations of camptothecin or its clinical analog topotecan as low as 10-20 nM (Fig. 4A), whereas non-amplified cell lines proliferated normally at these drug concentrations (Fig. 4B). To investigate in more detail the effect on proliferation and cell survival of camptothecin and topotecan in neuroblastoma cells, we carried out propidium iodide DNA staining and FACS analysis. In agreement with the MTT assay, in the presence of the drugs we observed a marked increase of cells blocked in the S or G2 phase of the cell cycle and an increase of fragmented, hypodiploid DNA, but only in cells with *MYCN* amplification. In contrast, non-amplified neuroblastoma cells showed a normal cell cycle profile in the presence of 10-20 nM camptothecin or topotecan (Fig. S1). Camptothecin and its analogues inhibit topoisomerase 1, therefore the observed effects could be explained if topoisomerase 1 expression were higher in *MYCN*-amplified, compared with *MYCN* non-amplified cell lines. However, the expression of topoisomerase-1 is highly variable among the different neuroblastoma cell lines and unrelated to the amplification status of *MYCN*, suggesting that the killing effect is independent from topoisomerase-1 expression (Fig. S2).

### The *B-MYB-MYCN* axis is disrupted by camptothecin and its clinical analogue topotecan

**Figure 5:**
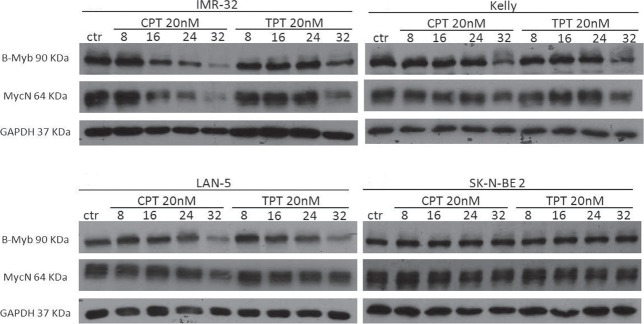
Effects of camptothecin (CPT) and topotecan (TPT) on the *B-MYB/MYCN* axis Western blot analysis showing the expression of B-Myb and MycN in the indicated neuroblastoma cell lines treated with CPT or TPT (20nM) for 8-16-24-32hrs. Expression of the housekeeping gene GAPDH was used as loading control.

**Figure 6:**
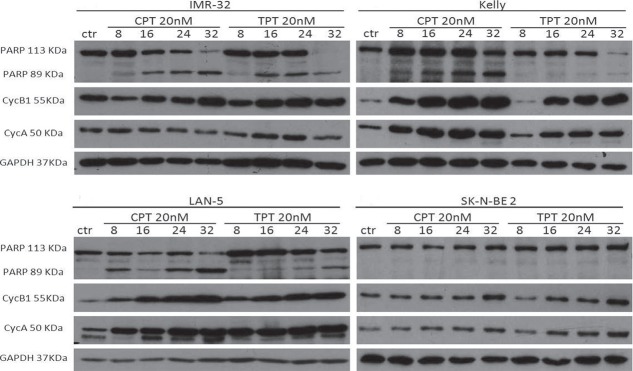
Effects of camptothecin (CPT) and topotecan (TPT) on cell cycle proteins and PARP cleavage Western blot analysis showing the expression of Cyclin A and cyclin B in the indicated neuroblastoma cell lines treated with CPT or TPT (20nM) for 8-16-24-32hrs. Cleavage of PARP is indicated by the appearance of a 89KDa fragment. The housekeeping gene GAPDH was used as loading control.

*B-MYB* and *MYCN* are engaged in a feed forward loop sustaining the expression of each other. Furthermore, B-Myb autoregulates its own expression.[38] Thus, we hypothesised that, by inhibiting B-Myb transcriptional activity, camptothecin and topotecan could also disrupt the expression of *B-MYB* and of its target gene, *MYCN*. Indeed, we observed that expression of B-Myb and MycN is downregulated by the drugs in 3 out of 4 *MYCN-*amplified cell lines (Fig. 5). The effect is not a mere consequence of reduced proliferation or cell death, since expression of cell cycle genes, such as cyclin A and cyclin B, or the housekeeping gene GAPDH is unaffected (Fig. 6). The increased expression of cyclin A/B by the drugs probably reflects the high number of cells blocked at the S and G2/M phases of the cell cycle (Fig. S1). We observed PARP fragmentation in drug-responsive cells, diagnostic of apoptosis (Fig. 6). Interestingly, camptothecin and topotecan did not cause inhibition of B-Myb/MycN expression and PARP cleavage in SK-N-BE2 cells. SK-N-BE2 cells contain mutated p53 [39] and it was previously observed that B-MYB expression is activated in cell lines with mutation of p53, perhaps explaining the drug-resistant phenotype [40].

### Forced expression of *B-MYB* confers resistance to camptothecin and topotecan in a *MYCN* amplified cell line

**Figure 7:**
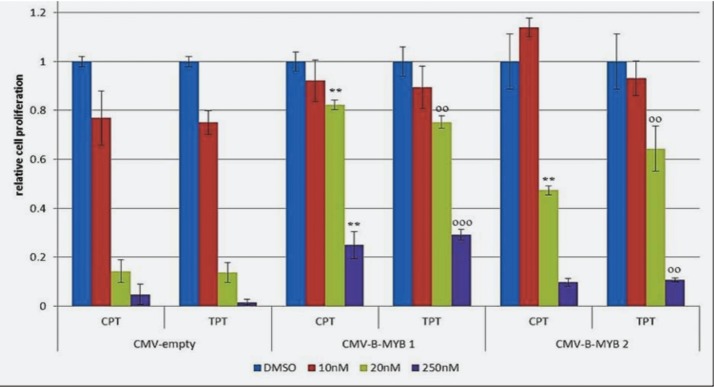
*B-MYB* rescues the killing effect of camptothecin (CPT) and topotecan (TPT) in *MYCN* amplified cells MTS proliferation assay showing the effect of CPT and TPT in LAN-5 cell clones transfected with pCMV-empty or pCMV-*B-MYB* plasmids. Bars indicate densitometric values relative to vehicle treated controls. Standard deviations is indicated by the error bars, statistical significance was assessed using the Student's T-test (*= p<0,05; **=p<0,01; ***=p<0,001; ^o^= p<0,05 ^oo^=p<0,01; ^ooo^=p<0,001;)

To demonstrate that the killing effect of camptothecin and topotecan in *MYCN*-amplified cell lines is caused by inhibition of B-Myb, we carried out a rescue experiment. To this end, we transfected LAN-5 cells with empty or *B-MYB* expression vectors. We then selected two clones expressing high levels of B-Myb (Fig. S3) and exposed them to 10-20nM topotecan or camptothecin. Notably, the inhibition of cell proliferation caused by the drugs was partially rescued by ectopic expression of *B-MYB* (Fig. 7). This experiment demonstrates that B-Myb is a key target of the drugs in neuroblastoma cells with amplification of *MYCN*.

Recent studies have revealed that B-Myb might promote cell cycle progression and cell survival by co-ordinating the expression of G2/M genes also by recruiting other transcription factors, such as FoxM1 [41]. *B-MYB* is overexpressed and amplified in a number of cancers, including breast, ovary, leukaemia and neuroblastoma and we have shown that neuroblastoma cells are addicted to *B-MYB* expression, but only in the context of *MYCN* amplification. B-Myb physically binds to the *MYCN* amplicon and is required for MycN expression. On the other hand, MycN promotes B-Myb transcription, feeding a positive loop that promotes survival of neuroblastoma cells [28]. Our new study shows that camptothecin and its clinically used derivative topotecan inhibit the transcriptional activity of B-Myb, inducing synthetic lethality in *MYCN* positive cells at dosages below the plasma concentrations attained in cancer patients undergoing treatment [42-44]. Thus, the molecular make up of neuroblastoma tumours is highly relevant to the sensitivity to camptothecin analogues. It will be interesting to verify whether the clinical response to these drugs is associated with the amplification status of *MYCN* and/or overexpression of *B-MYB* in neuroblastoma patients. If clinical studies will confirm this association, it is possible to envisage that paediatric patients with *MYCN* amplified tumours could be treated with lower dosages of the drugs, resulting in less toxicity and fewer long lasting sequelae.

## MATERIALS AND METHODS

### Plasmid vectors and transfections

The B-Myb responsive plasmid pGL2-mim1, containing five MYB binding sites, was constructed as follws: two oligonucleotides 5' CAA CGT TAT AGT GAG CTA AGA ACG TTA TAG TGA GCT AAG AAC GTT ATA GTG AGC TAA GAA CGT TAT AGT GAG CTA AGA ACG TTA TAG TGA GCT AAG C 3' and 5' TCG AGC TTA GCT CAC TAT AAC GTT CTT AGC TCA CTA TAA CGT TCT TAG CTC ACT ATA ACG TTC TTA GCT CAC TAT AAC GTT CTT AGC TCA CTA TAA CGT TGG TAC 3' were annealed and subcloned into the pGL2-promoter vector (Promega, USA). The pcDNA-*B-MYB* plasmid was obtained by subcloning the *B-MYB* cDNA into the pcDNA3.1(+) vector (Invitrogen, UK). Transfections were carried out using the Lipofectamine™ 2000 Reagent (Invitrogen, UK), following the manufacturer's instructions.

### Chemical screen and luciferase assays

The Diversity Set II (1364 compounds) and the Natural Product Set II (120 compounds) were obtained from the National Cancer Institute (NCI, Fisher BioServices, Rockville, MD). GIMEN, a *MYCN* non-amplified neuroblastoma cell line, was stably transfected with the pGL2-mim1 and the pcDna-*B-MYB* plasmids. Stably transfected GIMEN cells were exposed to each compound in 96 well plates for 24 h at a final concentration of 5 uM. After 24 hours, cells were subjected to luciferase assay and compounds causing over 50% inhibition of B-Myb transactivation in the absence of overt toxicity (i.e. rounding up and detachment of cells from plate) were used in validation experiments in which naïve GIMEN cells were exposed to the compounds after transient transfection of the *MYB* reporter and B-Myb expressing plasmids. Luciferase assays were performed with the Dual-Luciferase Reporter Assay System (Promega, USA), following the manufacturer's instructions, and assessed using a luminometer (BERTHOLD TECHNOLOGIES, Germany).

### Cell Lines

SH-SY5Y, LAN-5 and SK-N-AS cells were maintained in culture with DMEM supplemented with 10% heat-inactivated foetal bovine serum (FBS), 2mM l–glutamine, penicillin (100mg/ml), streptomycin (100mg/ml), sodium pyruvate (1mM) and non-essential amino acid (NEAA) (0,1mM).

IMR-32, GIMEN and Kelly cells were maintained in culture with RPMI 1640 supplemented with 10% heat-inactivated foetal bovine serum (FBS), 2mM l–glutamine, penicillin (100mg/ml), streptomycin (100mg/ml), sodium pyruvate (1mM) and non-essential amino acid (NEAA) (0,1mM).

SK-N-BE 2 cells were maintained in culture with 45% DMEM and 45% *Nutrient Mixture F12 Ham* supplemented with 10% heat-inactivated foetal bovine serum (FBS), 2mM l–glutamine, penicillin (100mg/ml), streptomycin (100mg/ml), sodium pyruvate (1mM) and non-essential amino acid (NEAA) (0,1mM).

SHSY5Y, SK-N-AS, SK-N-BE2, IMR32 and Kelly cell lines were purchased from the American Type Culture Collection, ATCC. LAN-5 and GIMEN cells were a kind gift of Dr. Mirco Ponzoni.

### MTS assay

The CellTiter 96® AQ_ueous_ Non-Radioactive Cell Proliferation Assay is a colorimetric method for determining the number of viable cells in proliferation or chemosensitivity assays. The CellTiter96® AQ_ueous_ Assay is composed of solutions of a novel tetrazolium compound (3-(4,5-dimethylthiazol-2-yl)-5-(3-carboxymethoxyphenyl)-2-(4-sulfophenyl)-2H-tetrazolium, inner salt; MTS) and an electron coupling reagent (phenazine methosulfate; PMS). MTS is bioreduced by cells into a formazan product that is soluble in tissue culture medium. The absorbance of the formazan at 490nm can be measured directly from 96-well assay plates without additional processing. The conversion of MTS into aqueous, soluble formazan is accomplished by dehydrogenase enzymes found in metabolically active cells. The quantity of formazan product as measured by the amount of 490nm absorbance is directly proportional to the number of living cells in culture.

### Cell cycle analysis

Cells were detached with 0.05% Trypsin-EDTA and collected by centrifugation at 2000rpm for 10 minutes. The cell pellet was resuspended and fixed in 3ml cold 70% ethanol for at least 30 minutes. After fixation, the cells were centrifuged at 2000rpm for 10 minutes at room temperature. The pellet was washed twice in 1ml PBS. During each wash, the cells were pelleted at 2,000 rpm for 10 minutes at room temperature. To ensure that only DNA is stained, cells were treated with 50μl ribonuclease A (RNaseA) solution (100μg/ml in PBS). Then 450μl of propidium iodide, PI (50μg/ml in PBS), was added directly to cells in RNase A solution. Cells were incubated for 30 minutes on ice. Samples were analyzed in PI/RNase A solution by a BD LSR II flow cytometer. All data for flow cytometry were analysed by FlowJo software.

### Western blot

Cells were lysates in RIPA Buffer (10 mM Tris-Cl (pH 8.0), 1 mM EDTA, 1% Triton X-100, 0.1% sodium deoxycholate, 0.1% SDS, 140 mM NaCl) and protease/phosphatase inhibitors (Roche). Proteins were separated by SDS/PAGE on 10% gels, transferred to poly vinylidene difluoride membrane (Amersham Pharmacia), and incubated with antibodies. Immunoblots were visualized by using the enhanced chemiluminescent system (Thermo).

### Antibodies

Monoclonal anti-B-Myb,[45] was kindly provided by Roger Watson. Other primary antibodies used were: anti-MycN, anti-Cyclin A, anti-Cyclin B1, anti-Cdc2, anti-PARP, anti-topoisomerase 1 (Santa Cruz Biotechnology) and anti-GAPDH (Cell Signaling). Horseradish peroxidase-conjugated secondary antibodies were purchased from GE Healthcare Life Sciences.

## Supplementary Figures


