# Supplementary material for: A chemical screen identifies the chemotherapeutic drug topotecan as a specific inhibitor of the B-MYB/MYCN axis in neuroblastoma
Source: Oncotarget. 2012 May 19;3(5):535–45. doi: 10.18632/oncotarget.498 (PMC3388183; doi:10.18632/oncotarget.498)
Supplement: Supplementary file 1 [file oncotarget-03-535-s001.pdf]

SOTTILE ET AL. A CHEMICAL SCREEN IDENTIFIES THE CHEMOTHERAPEUTIC DRUG TOPOTECAN AS A SPECIFIC INHIBITOR OF THE B-MYB/MYCN AXIS IN NEUROBLASTOMA.

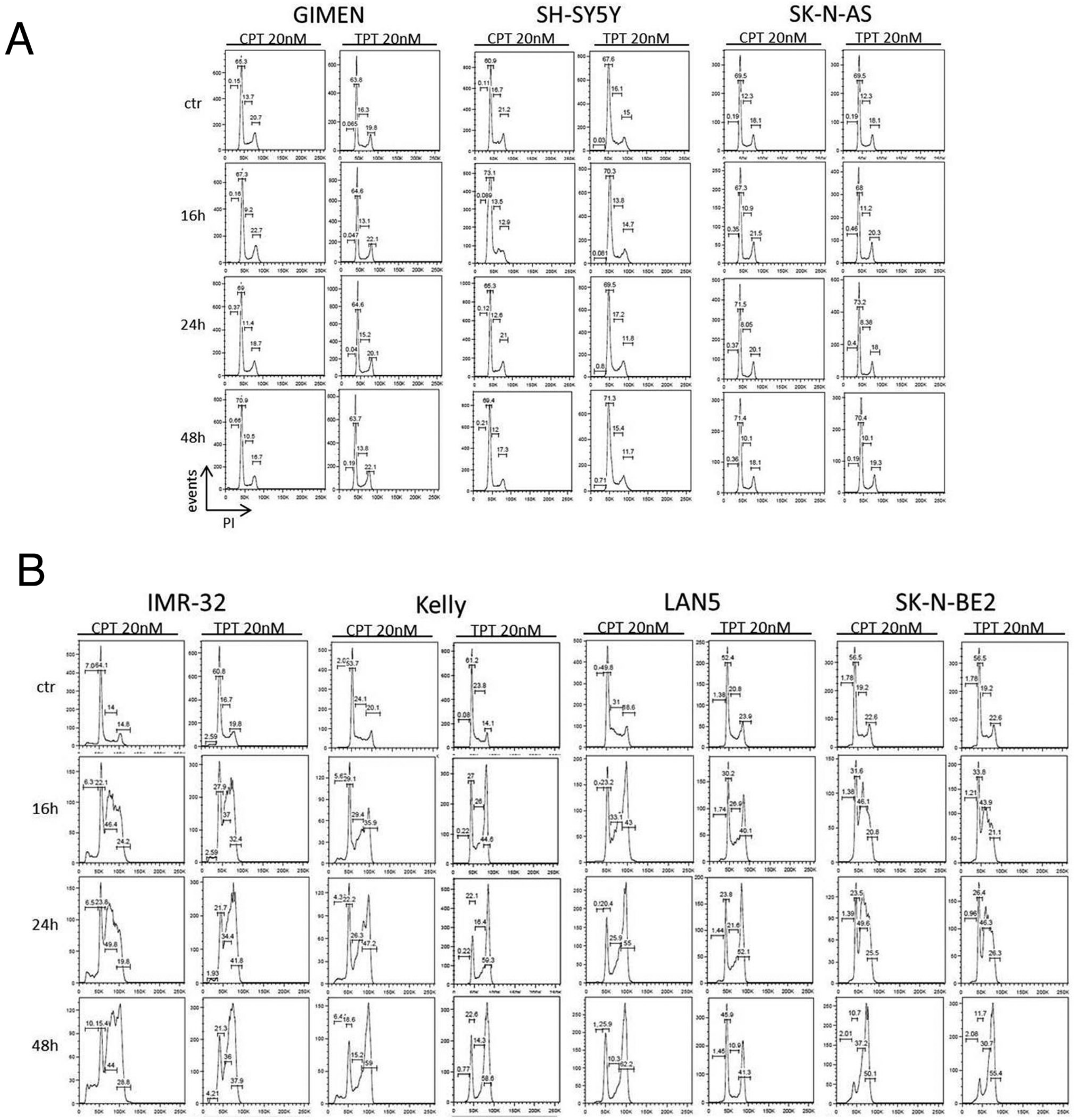

Supplementary Figure 2: Cell cycle analysis of neuroblastoma cell lines treated with 20nM camptothecin (CPT) or topotecan (TPT) and harvested at different time points, as indicated. The percentages of cells with hypodyploid DNA or in the dfferent phases of the cell cycle are indicated. (A) neuroblastoma cell lines without amplification of MYCN; (B) neuroblastoma cell lines with amplification of MYCN.

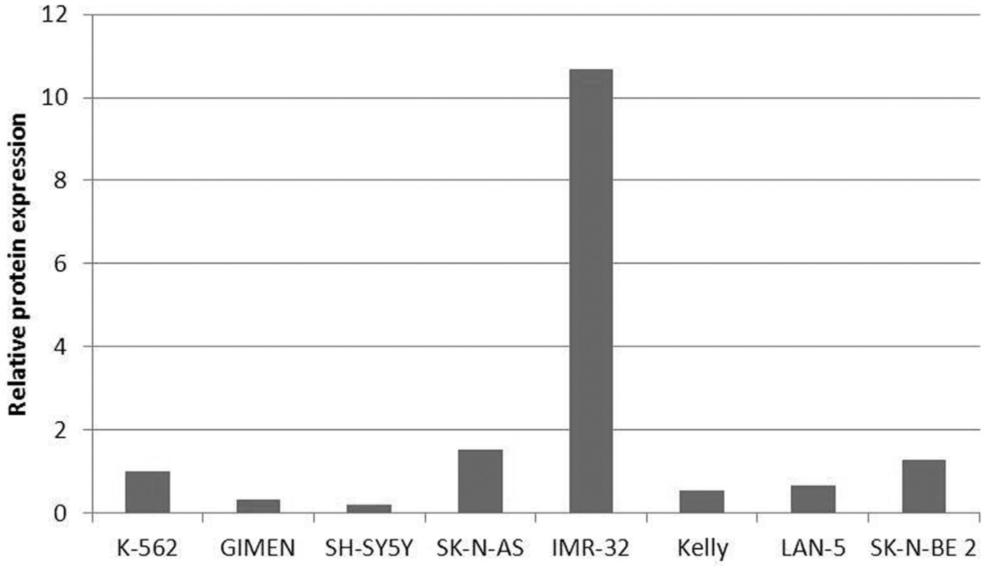

Supplementary Figure 2: Expression of Topoisomerase-1 in neuroblastoma cells. The expression of Topoisomerase-1 was quantified by western analysis with an antibody. The bars show the values of the densitometric units of topoisomerase-1 bands relative to GAPDH, used to normalize the expression. GIMEN, SH-SY5Y and SKNAS cells are MYCN negative cell lines. IMR-32, Kelly, LAN-5 and SK-N-BE2 are MYCN amplified cell lines. K-562 cells were used as positive control.

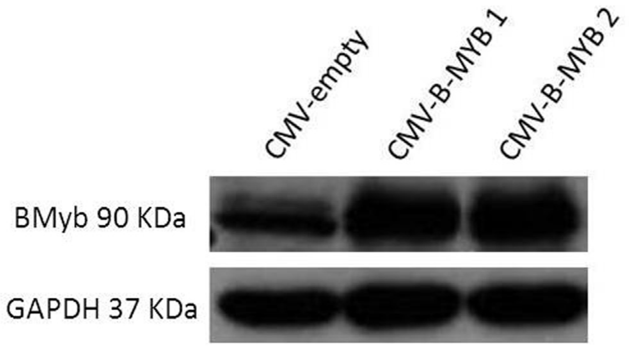

Supplementary Figure 3: Western blot analysis showing the expression of B-MYB in control or BMYB transfected cell lines.
